# Calling all archaeologists: guidelines for terminology, methodology, data handling, and reporting when undertaking and reviewing stable isotope applications in archaeology

**DOI:** 10.1002/rcm.8044

**Published:** 2018-02-08

**Authors:** Patrick Roberts, Ricardo Fernandes, Oliver E. Craig, Thomas Larsen, Alexandre Lucquin, Jillian Swift, Jana Zech

**Affiliations:** ^1^ Max Planck Institute for the Science of Human History Kahlaische Str. 10 D‐07745 Jena Germany; ^2^ McDonald Institute for Archaeological Research Downing St Cambridge CB2 3ER UK; ^3^ Archaeology University of York York YO10 5DD UK; ^4^ Leibniz‐Laboratory for Isotope Research Christian‐Albrechts‐Universität D‐24118 Kiel Germany

## Abstract

Stable isotope analysis has been utilized in archaeology since the 1970s, yet standardized protocols for terminology, sampling, pretreatment evaluation, calibration, quality assurance and control, data presentation, and graphical or statistical treatment still remain lacking in archaeological applications. Here, we present recommendations and requirements for each of these in the archaeological context of: bulk stable carbon and nitrogen isotope analysis of organics; bulk stable carbon and oxygen isotope analysis of carbonates; single compound stable carbon and nitrogen isotope analysis on amino acids in collagen and keratin; and single compound stable carbon and hydrogen isotope analysis on fatty acids. The protocols are based on recommendations from the Commission on Isotopic Abundances and Atomic Weights of the International Union of Pure and Applied Chemistry (IUPAC) as well as an expanding geochemical and archaeological science experimental literature. We hope that this will provide a useful future reference for authors and reviewers engaging with the growing number of stable isotope applications and datasets in archaeology.

## 1 INTRODUCTION

**Figure 1:**
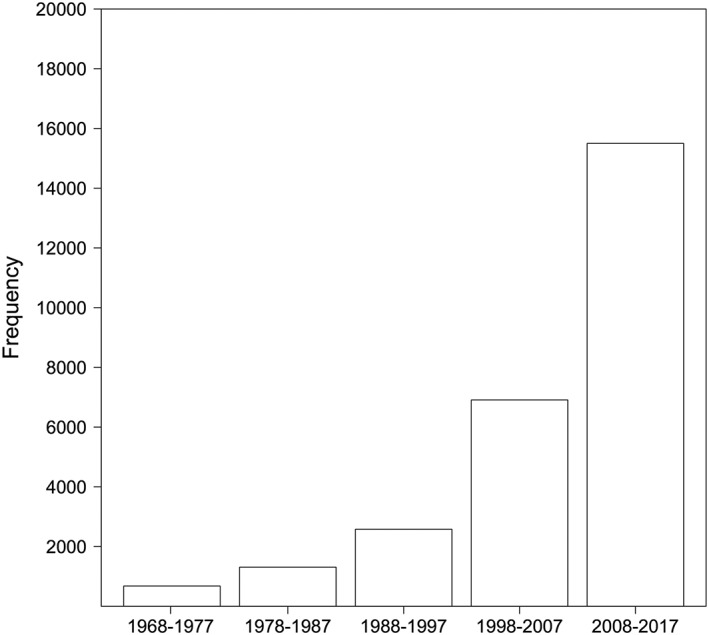
Bar‐plot showing the number of mentions of 'archaeology' and 'isotopes' in archaeological publications according to Google Scholar for the past five decades

The archaeological literature has seen an exponential increase in references to stable isotopes over the last half century due to reductions in equipment and sample processing costs, an increasing number of stable isotope laboratories and archaeological science units, and proliferating knowledge of archaeological science applications (Figure 1). Since some of the earliest archaeological applications in the late 1970s (e.g. 1), stable isotope analysis of human tissues, and of faunal and plant remains, have been used to study past diets, ecologies, and environments. More recently, stable isotope analysis of specific compounds has emerged as a more refined tool for studying ancient diet. For example, stable isotope analysis of individual amino acids isolated from bone collagen can be used to determine the proportion of marine versus terrestrial protein in the diet.^2^ The application of these methods to specific sites, periods, and regions of archaeological interest is increasingly commonplace and often led by archaeologists. While this democratization is certainly of benefit to the discipline, it comes with an enhanced responsibility on the part of archaeological users and reviewers. Commonly accepted guidelines in archaeology are essential to stimulate so‐called 'big data' approaches that allow data scientists and modelers to readily compile and access published data.

On the one hand, studies of pretreatment effects on stable isotope data,^3^, ^4^ the role of diagenesis in changing isotope ratios,^5^, ^6^ and understandings of ecological variability^7^, ^8^ are emerging from archaeologically focused laboratories in greater number. On the other hand, many papers appear without appropriate mention of basic measurement and calibration criteria (see Szpak et al^9^ for an estimate of the scale of this problem), quality control, justification of pretreatment selection or sampling, full presentation of methods and datasets, and with inappropriate use of graphs and statistics. Here we seek to disseminate, in Open Access form, best practices in this regard relating to the bulk stable carbon and nitrogen isotope analysis of organics; bulk stable carbon and oxygen isotope analysis of carbonates; single compound stable carbon and nitrogen isotope analysis on amino acids (AA) isolated from collagen and hair keratin, and stable carbon and hydrogen isotope analysis of fatty acids from artefacts, bone, and sediments based on IUPAC^10^ recommendations and an expanding geochemical and archaeological science literature. While some reviews have touched on a few of these themes in the context of geochemistry as a whole,^11^, ^12^ and more recently in forensics,^13^ we have written this article to directly increase information flow to archaeological science practitioners, students, and reviewers less familiar with these techniques. We hope this will ensure that archaeologists continue to make substantial contributions to cross‐disciplinary advancements in mass spectrometry methods and applications.

## 2 A NOTE ON TERMINOLOGY

Tyler Coplen^11^ has previously provided a thorough discussion of stable isotope terminology in *Rapid Communications in Mass Spectrometry*. However, many archaeological isotope publications use incorrect terminology, some of which has also been highlighted by Zachary Sharp for geochemistry in general.^14^ Firstly, the isotope ratio for samples and standards obtained from Isotope Ratio Mass Spectrometers is in the form of ^13^C/^12^C, ^15^N/^14^N, and ^18^O/^16^O. The corresponding *δ* ^13^C, *δ* ^15^N, and *δ* ^18^O values are not ratios *per se* but rather values produced from an equation that relates the measured isotope ratio of a (given) sample to the isotope ratio of a standard reference material (SRM):

(1) $$\delta^{i}E_{\text{sample}}=\frac{\left(\frac{i_{E}}{j_{E}}\right)\text{sample}-\left(\frac{i_{E}}{j_{E}}\right)\text{reference}}{\left(\frac{i_{E}}{j_{E}}\right)\text{reference}}$$

The delta notation (δ^i^E_sample_), defined through Equation (1), is commonly employed in reporting stable isotope results. For a certain chemical element (E) the ratios of the abundances of the heavier (i) versus lighter (j) isotope are measured in a sample ( EiEjsample) and in a SRM with an internationally accepted δ value ( EiEjreference).^15^ Equation (1) expresses the relative difference in absolute isotopic abundance ratios of a sample against the chosen SRM. Per mil notation (‰) is used as a convenient means of reporting small numerical values.^16^ This is not a SI unit of measurement; it is simply a unit of comparison of the sample with a standard with an internationally recognized isotopic abundance.^11^, ^15^

It is correct to state that an archaeological material such as bone collagen has a "stable carbon isotope composition". However, it is not possible for bone collagen to have a *δ* ^13^C, *δ* ^15^N, *δ* ^13^C_AA_, *δ* ^15^N_AA_, or *δ* ^18^O 'composition' or 'signature'. As Sharp^14^ points out, *δ* values are numbers and "a composition of numbers has no meaning". Similarly, a signature is something that is individualized and constant.

Example: **Not**: "*The δ* ^*13*^ *C composition/signature of the bone collagen was = –20.3 ‰*" **But:** "*The δ* ^*13*^ *C value of the bone collagen was –20.3 ‰*".

There are often also terminological issues when comparing different samples or measurements during the reporting of results or interpretation. The term 'isotopically depleted' is inappropriate when referring to *δ* values of archaeological materials. This is not only due to the vagueness of this term (as is also the case with discussing an 'isotopic composition' or 'isotopic value' above), but also because a sample of collagen, plant, or carbonate is not depleted or enriched in isotopes generally. Instead the terms depleted or enriched should be reserved for changes in the proportion of the heavy isotope (e.g. ^13^C) of the element in a given substance or fractionation process.

Example: **Not:** "*The δ* ^*18*^ *O value of the cattle teeth is enriched compared with that of the pig teeth*" **But:** "*The cattle tooth enamel was more* ^*18*^ *O‐enriched than the pig teeth*".

Similarly, it is not possible to have heavier or lighter *δ* ^13^C, *δ* ^15^N, *δ* ^13^C_AA_, *δ* ^15^N_AA_, or *δ* ^18^O values. This refers to the isotopes within the ratio that was measured. *δ* ^13^C, *δ* ^15^N *δ* ^13^C_AA_, *δ* ^15^N_AA_, and *δ* ^18^O can either be more negative or positive or lower or higher. Something is also not 'isotopically negative'. The terms 'more negative' and 'more positive' in reference to delta‐notated values are passable, but the use of high/higher and low/lower is more appropriate terminology for the comparison of numbers.

Example: **Not**: "*Humans from later periods had heavier δ* ^*13*^ *C and δ* ^*15*^ *N values than those from earlier periods*" **But**: "*Humans from later periods at the site have higher δ* ^*13*^ *C and δ* ^*15*^ *N values than those from earlier periods*".

The use of a standard appropriate terminology aids clarity in publications that can often use a multitude of different terms when referencing *δ* values.

## 3 ACKNOWLEDGING DIAGENESIS AND SELECTING A PRETREATMENT

The potential for variability in burial environment (e.g., humidity, pH, microbial attack, temperature and time) to alter the *in vivo* isotope values of bone collagen, bone bioapatite, dentine collagen, and tooth enamel bioapatite has been documented in archaeological applications since the 1980s.^17^, ^18^, ^19^, ^20^ Furthermore, the mechanisms behind these changes, especially for tooth enamel bioapatite and bone collagen, are relatively well known.^5^, ^18^, ^21^ While this has led to basic diagenetic checks that are applied in many archaeological science publications (Table 1), this information often goes unpublished or is not utilized even where burial environments are problematic.

**Table 1:** Summary of diagenetic checks and available, previously utilized methods for different archaeological materials

Sample type	Common checks	Other methods
Bone collagen	%C, %N, C:N atomic ratio, % collagen yield	***FTIR* –** Bone collagen content ***Raman spectroscopy** – Bone collagen content* ***GC/MS –*** Amino acid profiling
Dentine collagen	%C, %N, C:N atomic ratio, % collagen yield	
Collagen amino acids	GC‐FID or GC/MS to assess impurities and compare amino acid profiles to modern reference samples of the same taxa	
Fatty acids	GC and GC/MS to assess sample quality and lipid yield	
Crop remains	%C, %N, C:N atomic ratio	***FTIR*** – check for the presence of carbonates and humic acids
Tooth enamel bioapatite	%CO_3_, expected δ^13^C range according to species and region (e.g. grazers vs non‐grazers)	***FTIR*** – check for calcite, changes in crystallinity parameters (API, BPI, IRSF, BAI) ***Microscopic luminescence*** – transferal of metallic elements across material boundaries ***Measurement of trace elements***– bulk values or section profiles
Bone bioapatite	%CO_3_, δ^13^C pattern between grazers and non‐grazers	***FTIR*** – check for calcite, changes in crystallinity parameters (API, BPI, IRSF, BAI) ***Microscopic luminescence*** – transferal of metallic elements across material boundaries ***Measurement of trace elements*** – bulk values or section profiles
Terrestrial snail shell	%CO_3_	***Light microscopy, scanning electron microscopy, X‐ray diffraction, and FTIR*** – check for conversion from aragonite to calcite and presence of secondary calcite.
Marine shell	%CO_3_	***Light microscopy, scanning electron microscopy, X‐ray diffraction, and FTIR*** – check for conversion from aragonite into calcite and presence of secondary calcite.

Restrictions of cost, equipment availability, training and sample size availability, and the need for continued development mean that we do not recommend routine application of complex methods, particularly for bone collagen and tooth enamel bioapatite in relatively well‐known environments during the Holocene and even the Late Pleistocene. However, there are materials for which regular diagenetic assessment is essential. For example, Loftus et al^22^ recently demonstrated a rapid way of using Fourier‐Transform Infrared Spectroscopy (FTIR) to determine whether marine shell calcite had diagenetically converted into aragonite. They documented that this can occur locally, altering *in vivo δ* values, making the testing of sub‐samples essential in the sequential analyses of this material.

Similarly, charred crop remains are increasingly isotopically analyzed in order to provide insights into growing conditions, climate change, and farming strategies. It has been documented that in some cases burial environments can modify pre‐burial isotopic ratios of charred grains.^23^ Thus, it is recommended that a preliminary study is carried out relying on a sub‐sample to compare the isotopic ratios of untreated and pre‐treated charred grains.^24^ In many instances pre‐treatment may not be necessary, allowing for a greater sample throughput and reduction in operating costs, but this should be checked when the method is being applied to new burial environments.^24^

Elemental compositions and collagen yields are regularly reported in the context of collagen preservation, and the percentage carbonate reported for tooth enamel. We would recommend more in‐depth diagenetic studies over longer time periods, such as the Miocene, Pliocene, and Early Pleistocene, and in challenging taphonomic environments where these materials have been less well studied, such as those with high water throughput or potentially high trace element abundance. FTIR, Fluorescence Microscopy, and X‐ray Fluorescence (XRF) or Inductively Coupled Plasma Mass Spectrometry (ICP‐MS) measurements of trace elements have all proved promising in this regard.^5^, ^6^

Diagenesis is an important problem to address. However, it is also important to note that although studies might document diagenetic alteration of isotopic ratios in certain archaeological materials and contexts (e.g. 5), this does not mean that the same materials should not be analyzed in other settings.^25^, ^26^, ^27^ This has been a particular problem in stable carbon and oxygen isotope studies of tooth enamel, which have only recently begun to expand in archaeology following a period of distrust. Opinions of the utility of different materials for stable isotope analysis need therefore to be formed on the basis of the wider, current literature rather than a few papers and preconceptions.

It is also important for researchers and reviewers to probe the impacts that different pretreatment protocols might have on a sample's material structure and *δ* value.^3^, ^23^, ^28^ A plethora of different pretreatment techniques are still being used for the isotope analysis of bone collagen, bone bioapatite, tooth enamel bioapatite, tooth dentine, and plant remains, impacting the reliability of cross‐comparison (e.g. 28). In many cases, such as for tooth enamel bioapatite and bone collagen, the effects of different techniques are minimal.^3^, ^29^ However, choices of pretreatment for bone bioapatite,^3^, ^28^ shell and soil carbonate,^30^ and crop remains^23^ can be more significant in attempts to replicate the *in vivo δ* value. In these latter cases, it is not unreasonable for a reviewer to request information regarding sample pretreatment and sample preparation if this has not already been provided.

**Figure 2:**
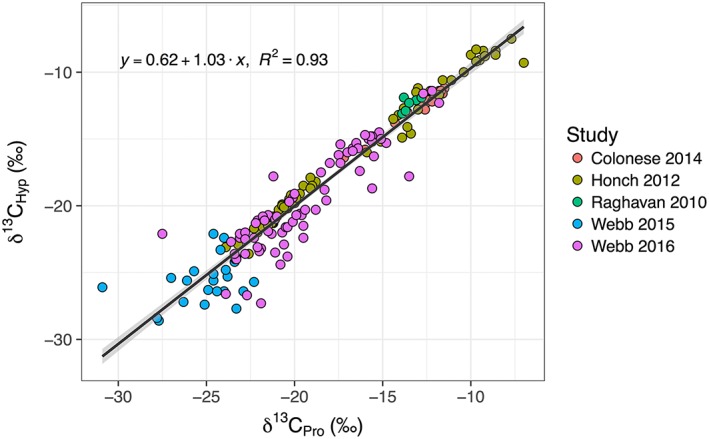
Comparison of published collagen and dentin proline and hydroxyproline *δ* ^13^C values from archaeological materials shows that are only minor offsets between these amino acids (y = 0.62 + 1.03x, adjusted R^2^ = 0.9252, F(1,184) = 2290, P <0.001). The data were obtained from five studies that used LC/IRMS.^34^, ^35^, ^36^, ^37^, ^38^ The solid lines depict the linear correlations, and the shaded areas depict 95% confidence intervals [Color figure can be viewed at wileyonlinelibrary.com]

Diagenetic and pretreatment biases for compound‐specific approaches remain relatively under‐explored and un‐reported in the context of archaeological science. For the stable isotope analysis of proteinogenic amino acids, similar measures to those employed in bulk isotope analyses of bone collagen are used to assess diagenesis (Table 1). When evaluating amino acid *δ* ^15^N data, there should be only minor differences between proline (Pro) and hydroxyproline (Hyp) *δ* ^15^N values in collagen since the nitrogen in Hyp derives from Pro.^31^ Similarly, the expected slope for the *δ* ^13^C_Hyp_ value as a function of the *δ* ^13^C_Pro_ value is 1 because, after formation of immature collagen, Hyp is synthesized exclusively from Pro.^32^, ^33^ Empirical evidence from five archaeological studies of mammal bone collagen samples shows that Hyp (y) and Pro (x) *δ* ^13^C values usually follow a 1:1 line (y = 0.9239x − 1.4745, R^2^ = 93.6%, n = 171) thus lending credence to using the relationship between Hyp and Pro *δ* ^13^C values for data quality control (Figure 2).

With regard to pretreatment biases, it is important to homogenize samples before analysis by milling or grinding, particularly for composite materials. After the subsequent acid hydrolysis we recommend filtering out particles from the digest using glass fiber or inert membranes. For complex or composite samples, amino acids should be separated from other compounds in the digests via ion‐exchange chromatography. This procedure may induce isotope effects on certain amino acids as reported previously in the literature.^39^ For this reason, it is recommended that each lab investigates whether their cleaning protocols have isotope effects in order to correct for these offsets. For lipids from artefacts, bone, and sediments, the normal practice is to screen the samples with gas chromatography (GC) and gas chromatography mass spectrometry (GC/MS) to verify the compounds of interest and check for co‐elution and contamination (Table 1). Contaminants, such as from plastic sample bags, should be reported and accounted for. Negative controls involving blank extractions and extractions of associated sediments and pottery should be routinely performed. Similarly, amino acid chromatograms should be checked for co‐elution from non‐proteinogenic compounds.

## 4 SAMPLING

Problems of diagenesis and taphonomy (e.g. funeral practices) are also the primary cause of an inevitable issue in archaeological science stable isotope applications: low or variable sample size. The nature of archaeological preservation makes it very difficult to be prescriptive in sample size necessity in different isotopic applications. However, it is important that both the variability within a given sample (e.g. a single bone) and the variability within the population under study (e.g. a sample of the same skeletal element from a series of different individuals) are taken into account when interpretations relating to δ differences between groups are being made. Furthermore, if a confident difference is to be asserted, the sample size must be large enough for the chosen statistical test to operate effectively.

In order to determine variability within a given type of sample for a particular study we recommend a pilot study measuring at least three repeat aliquots that are extracted and pretreated separately from a single sample (e.g. a bone). The measurement standard deviation for these extracts will provide a useful evaluation of *δ* uncertainty resulting from burial environment, pretreatment, and natural heterogeneity in a sample. It is especially important to do this when an archaeological material is being analyzed for the first time in a particular burial environment. Suitable statistical testing, that meets sample size requirements, will address the problem of whether variability within a given population is greater than the variability between populations. Where only small sample sizes are available for a context, care should be taken when comparing the data with that from other contexts.

## 5 CALIBRATING ISOTOPE DATA

Perhaps one of the most significant omissions in stable isotope applications in archaeology is the proper reporting of the stable isotope abundance measurements of standards and reference materials used in the analysis, together with their adopted values and uncertainties.^15^ Szpak et al^9^ recently found that a large majority of archaeological isotope studies do not provide adequate information regarding isotope measurement calibration and analytical uncertainty. Isotope Ratio Mass Spectrometers generate raw *δ* values using a single‐point calibration relative to a laboratory working gas, the composition of which is arbitrary and can change over time.^40^ The working and sample gases are introduced into the isotope ratio mass spectrometer using two different systems. This means that the working gas does not undergo the same physical and chemical processes as those affecting the sample gas, thus voiding the important principle of 'Identical Treatment' that must hold for calibration materials.^41^

To make the raw *δ* values meaningful for cross‐study comparison and interpretation, it is essential that the measurements be calibrated to internationally accepted *δ* scales, using standard reference materials (SRMs) with known isotopic ratios interspersed among samples in each analytical sessions.^41^, ^42^ Following Coleman and Meier‐Augenstein^12^ and Coplen,^11^ and established IUPAC^10^ requirements for data calibration in isotope geochemistry more generally, authors should:

- Express *δ* values relative to current international measurement standards. References to these standards should also avoid using redundant standards such as SMOW (it is now VSMOW) or PDB (it is now VPDB);^11^, ^15^
- Where a second international measurement standard is used to define the range of the *δ* scale, for example SLAP (SLAP2) water for δ^2^H and δ^18^O measurements, normalize *δ* values using both standards and state this clearly;
- Report *δ* values of SRMs with known, internationally accepted isotopic ratios (previously calibrated to VPDB, AIR, or VSMOW); at least two calibration SRMs, such as USGS40 or USGS41a^43^, ^44^ in the case of *δ* ^15^N and *δ* ^13^C values, should be used to anchor the raw sample isotopic ratios at the high and low ends of the isotopic range, enabling shifting and stretching onto an international scale via a 'two‐point' calibration^41^, ^45^, ^46^ (Figure 2).

Most archaeological science applications of isotope analysis meet requirements i) and ii) (where required). However, the names and *δ* values of calibration SRMs, and the process of normalization used for iii), are often lacking (see Szpak et al^9^). For example, sometimes only one calibration SRM is used, making the appropriate stretching of the data impossible.^9^, ^41^ Inadequate reporting may be the result of archaeologists sending their samples away to a commercial chemistry lab that provides 'final' values and no insight into the process of measurement, normalization, or the standards that were run alongside the samples. However, the necessary information must be requested and verified from commercial labs prior to the sending of samples. Notably, laboratories with ISO/IEC 17025^47^ "General Requirements for the Competence of Testing and Calibration Laboratories" (see clauses 4.13.2.1 and 5.10) accreditation must provide the client with all records and documentation pertaining to the analysis of the client's samples including raw data, data calibration, and data evaluation.

Archaeological science authors and reviewers should pay close attention to the selection and reporting of the utilized calibration SRMs chosen to 'bracket' the expected *δ* range of the samples. If internal SRMs are used, it is necessary to specify their accepted values, and how these values were obtained relative to internationally accepted standards.^12^, ^41^, ^42^ Calibration SRMs should also be 'matrix‐matched', as far as possible, to the samples under study. In other words, they should have a similar elemental composition and structure to the samples. The number of calibration SRMs used in a study should also be stated. Szpak et al^9^ recommend that at least 10% of the total analyses should be calibration standards.

For compound‐specific isotope analysis, check standards should be composed of a mixture of compounds of interest (e.g. palmitic and stearic acid methyl esters) and of other stable compounds eluting closely (i.e. eicosane), corresponding to the expected range of isotopic value (see Brand et al^15^). SRMs with known isotope ratios that bracket the expected isotope range should also be run for calibration purposes (e.g. 48). Ideally, multiple compounds found within the sample should be used as SRMs. Finally, internal reference compounds can also be included in each analytical run to help with calibration or precision estimation.

## 6 QUALITY CONTROL AND QUALITY ASSURANCE

**Figure 3:**
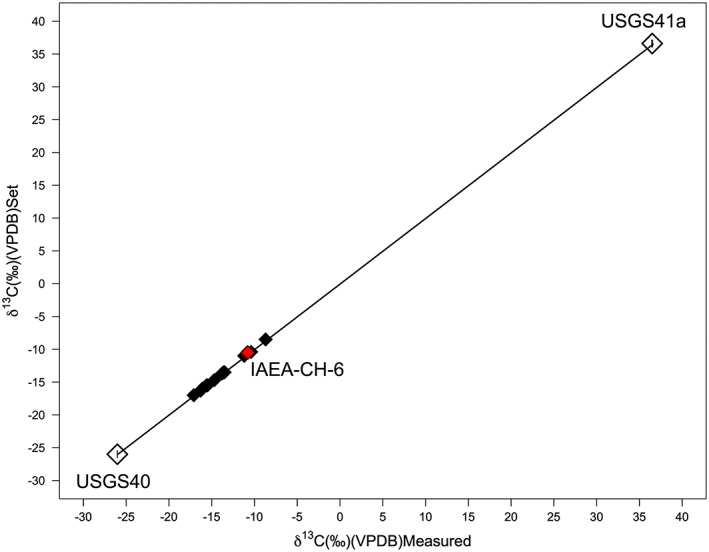
Plot demonstrating data normalization using secondary measurement standards [Color figure can be viewed at wileyonlinelibrary.com]

It is also important to document systematic errors, such as instrumental drift, related to the analytical accuracy of a system, as well as the relationship between the *δ* value measured and the true *δ* value of the sample. This should be assessed using check SRMs that are not used for calibration. These are treated as having 'unknown' values just like the samples (Figure 3). As with the calibration SRMs these should be matrix‐matched to the samples. While Szpak et al^9^ recommend that 10% of the total analyses should be 'check' standards and placed at regular intervals within a session, the exact proportion will depend on the accuracy required for a given application. The *δ* values of these check SRMs (mean ± 1σ) for each measurement session should be reported, even if only in supplementary information.^10^ We recommend that these records of accuracy be kept up‐to‐date and used as an ongoing measure of laboratory performance that can even be publicly displayed.

We would recommend using more than one check SRM to test whether this magnitude is similar across slightly different materials. Furthermore, tests for thresholds of linearity effects (whereby IRMS‐based techniques return different *δ* values depending on the mass of sample) should be made for each new material or SRM run on an IRMS system.^13^ This all helps to assess the discrepancy between measured *δ* values and 'true' sample isotope composition within a study, and ensure that interpretations of the data are balanced accordingly. If this systematic error is related to instrumental drift, for example in the form of temperature or humidity changes affecting the IRMS system, and is shown to be continuous across the entire analytical session, drift corrections can be made using check SRMs placed at regular intervals throughout the session.^9^, ^41^ If these corrections are made details should be provided in the text or supplementary information.

Precision refers to the repeatability or reproducibility of measurements and random errors rather than systematic errors. This can be assessed for a given study using either check or calibration SRMs, as well as repeated measurement of samples. We recommend reporting duplicate measurements of the same aliquots of archaeological samples where possible. The precision of repeated SRMs or samples should be reported as the mean ± 1σ. As Szpak et al^9^ note, it is essential that matrix‐matched SRMs are used when precision estimates are used to validate interpretation. For example, a precision of *δ* ^13^C measurements of ±0.01 ‰ may be attainable with a pure carbonate reference material. However, this is almost meaningless when provided for a study focused on tooth enamel (*ca* 3–10 % carbonate) sample *δ* ^13^C values, on which a precision of ±0.01 ‰ is impossible on even the most refined systems as a result of natural inhomogeneity. It is often best to use in‐house reference materials, or repeat sample aliquots, for a more realistic assessment of precision in archaeological science applications.

Precision measured through repeat analyses is not necessarily the best overall measure of uncertainty in sample *δ* values, however.^49^ A better measure is obtained by propagating errors across the whole process of sample selection, preparation, measurement, and normalization. This can be done using a 'bottom up' approach (e.g. 49, 50) or via a 'top down' approach.^13^ Together, these criteria enable archaeological science‐focused laboratories to validate their pretreatment and instrumental methods more widely, the importance of which has recently been emphasized in forensic studies.^13^ Given the growing importance of data compatibility between laboratories, and over long time scales for archaeological interpretation between populations and sites, it is essential that each study and laboratory demonstrate that its methods of sample selection, pretreatment/extraction, measurement, and calibration meet accepted criteria.

**Figure 4:**
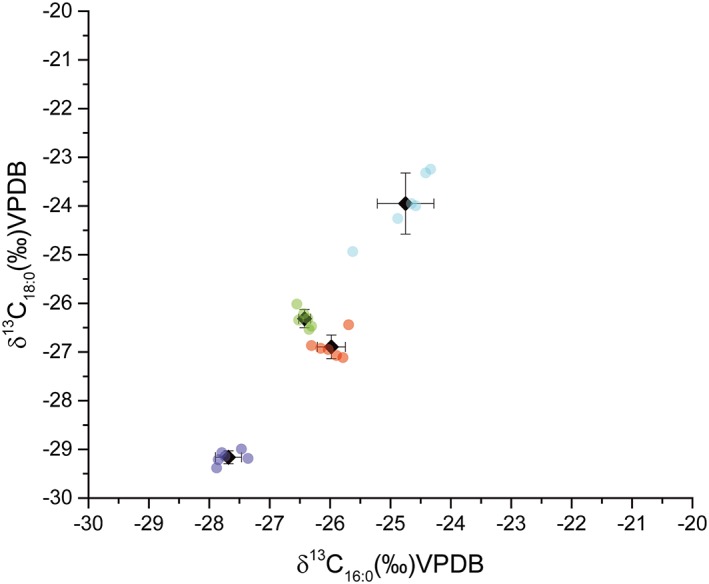
An example of intra‐laboratory variability in compound‐specific stable isotope measurements. Four pottery sherds were sampled by drilling and the resulting powder homogenized. Aliquoted subsamples were distributed to three different analysts at the University of York and extracted in duplicate according to established protocols.^51^ Plot shows the *δ* ^13^C values of palmitic (C_16:0_) and stearic acid (C_18:0_), following calibration and correction for derivatization, for each separate extract and the mean and standard deviation of each sherd. Each sherd is represented by an individual color [Color figure can be viewed at wileyonlinelibrary.com]

In the case of a given study, we recommend the analysis of an SRM with a known isotope *δ* value and treating it alongside samples to establish the degree to which treatment and measurement causes sample *δ* values to deviate from their 'true' value. This is particularly important in single‐compound approaches. For example, in Figure 4 we show the results of *δ* ^13^C analyses of fatty acids repeatedly extracted from homogenized pulverized pottery sherds to assess within‐lab reproducibility. The uncertainty achieved through this exercise (±0.6 ‰ 1σ) was greater than obtained from repeated measurements of a single extract (typically ±0.3 ‰ 1σ), emphasizing the importance of propagating errors beyond measurement precision and the importance of extracting a SRM 'sample' during extraction. It is also particularly important when reporting this procedure to account for the addition of C and H during the derivatization process. As a result, the isotope ratios of reference compounds should be reported before and after derivatization with measurement uncertainties.

In the case of inter‐laboratory validation, inter‐laboratory comparisons should be designed to take into account the points raised above relating to uncertainty arising from pretreatment, calibration, and standard use, in order to enable laboratories to critically identify the largest sources of errors during sample cleaning, extraction, and instrument calibration. Until now this has been rarely undertaken, or has been published without suitable determinations of analytical uncertainty (e.g. 52), particularly in the case of single‐compound analysis. Swapping and repeated extraction of samples between laboratories is therefore recommended in order to document the precision of a given archaeological measurement. In addition, for amino acid and fatty acid stable isotope analysis we propose an anonymous, blind inter‐laboratory study of the *δ* values of two calibrated mixtures known by the coordinator. For AA analysis it would also be useful to include a matrix sample of bone collagen already measured for bulk *δ* ^13^C and *δ* ^15^N values.

## 7 LEAVING A FULL PICTURE: METHOD AND DATA AVAILABILITY

Another potential problem in archaeological science applications of isotope methods is the omission of the full methodological details and datasets in publications. While most papers focused on bulk isotope measurements also provide method information that could be practically followed by any researcher seeking to emulate the study, these data are often missing when isotope data is provided as supplementary information to radiocarbon dating. *δ* ^13^C and *δ* ^15^N values are often presented for bone collagen samples that have been radiocarbon dated, but information relating to their preparation, normalization, and comparison with reference materials should also be provided so that it can be determined whether these results are useful for subsequent stable isotope comparisons.

Full data reporting is also essential in publications. This is, in part, because it is useful to have datasets available for comparison with existing literature, particularly given the rise of 'big data' approaches also within archaeology.^53^ One of the most common oversights in this regard is the production of a mean and standard deviation plot for a given human group or faunal group as a useful, simplified graphical representation of one's data 'average' and 'spread'. Nevertheless, in some cases the corresponding data table will also only report the mean and standard deviation for that group without an additional table that includes the individual measurements or samples that comprise that group. It should be said that this is relatively rare, although often things such as associated fauna can be treated as "background data" and not reported.

A paper relying on data that is not fully provided should not make it past review. Similarly, where modern samples have been incorporated as a useful analogy, collection locations, growing conditions (for plants), and local climate or environmental data should be reported. The date of sample collection is particularly important so that the appropriate atmospheric or oceanic *Suess effect* can be applied.^54^ If *Suess effect* corrections are made these need to be explicit and include appropriate uncertainties. In general, all calculation and correction stages applied to raw data should be documented in a publication and its associated tables for transparent evaluation. Where groups are compared, full archaeological context information, and the logic behind such groupings, should be provided.

Transparent methodology is also often missing in many compound‐specific isotope papers, hindering replication and application beyond select groups. Given the rapid development of these specialist approaches and the ample space available in supplementary information sections this should be remedied. There is also a growing problem of data reporting in more recently applied compound‐specific approaches. Here studies must provide the final *δ* value for each sample (mean and analytical standard deviation) across replicate injections or include chromatograms of representative samples.

One potential solution to these problems is a central repository for archaeological isotopic data, and calls for this have been put forward.^55^, ^56^, ^57^ Central data repositories, such as GenBank, exist for the field of archaeogenetics and proteomics.^58^, ^59^ However, it is also important to notice that applications of stable isotope data are extremely diverse and different research fields have specific data requirements. Partnership‐based initiatives, such as IsoMemo, attempt to bring together multiple repositories of stable isotope data from archaeology, ecology, and environmental sciences.^60^ The goals of the initiative are to coordinate data collection efforts, sharing and centralization of data, creating tools (e.g. user‐friendly graphical interfaces) for facilitating data access, building interdisciplinary projects, and establishing common data standards.

The latter includes, for instance, adoption of common terminologies and the assignment of unique codes to stable isotope labs and for reported measurements. Among IsoMemo partners are stable and radiogenic databases devoted to the storing of archaeological data from varied regions and time periods (e.g. 61, 62, 63) although there is still a general lack of awareness of their availability. While data collection requires the overcoming of political and data‐retention concerns, it seems reasonable that for archaeological science isotope papers to be published all reported data should either be placed in a similar repository or made fully available in a table within or attached to the publication. Journals should also consider changing their requirements for table formatting in some cases, as .xls or .csv format makes for greater ease of re‐use of the data than PDF forms.

## 8 GRAPHS AND SCALING

The development of open access graphical and statistical analysis programs makes sophisticated plotting tools widely available. However, there are several caveats to keep in mind when selecting appropriate methods for data display. First, a graph should be chosen that fits the data at hand: for normally distributed data a mean and standard deviation plot are appropriate when summarizing the dataset. Here the sigma of the standard deviation should be specified, as well as the range of the sample distribution that it describes (i.e. 68 % or 95 %) when interpretations are made. For non‐normally distributed data, box plots would better summarize the data. Similarly, choices of data groupings (e.g. species, period, or stratigraphic layer) should be fully justified and considered in the text. For example, plotting humans from one period/site and fauna from another period/site may be the only option left to the researchers, but this should be explained clearly.

**Figure 5:**
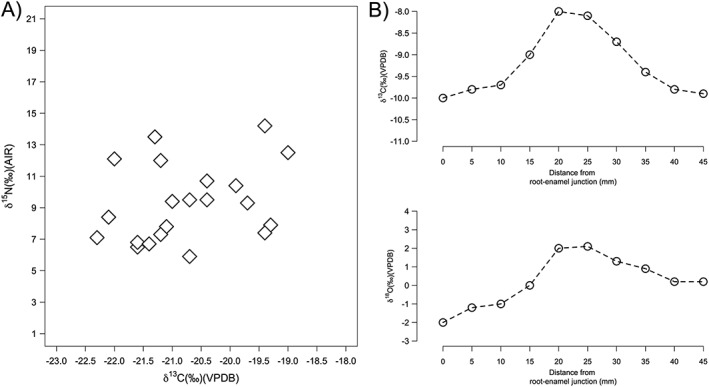
A) Example of differential scaling of *δ* ^15^N and *δ* ^13^C values in a scatterplot. B) Example of differential scaling in a sequential plot of *δ* ^13^C and *δ* ^18^O values of tooth enamel

There can be interpretive issues with the use of axes scales in terms of both scale length and scale divisions in bi‐plots of two isotope parameters, the most common being *δ* ^13^C values plotted against *δ* ^15^N values for bone collagen, dentine collagen, or crop remains. Variation in ‰ in a given human or ecological system may be greater for *δ* ^15^N than *δ* ^13^C values, and vice versa. *δ* ^15^N, *δ* ^13^C, and *δ* ^18^O values will also vary significantly between sites, periods, and ecological systems. These factors may lead to decisions to adopt different scale lengths and divisions for different isotopic parameters or archaeological periods and sites (Figure 5A). However, if this is done, researchers must be wary of statements such as: "Figure 5A shows that the variation in *δ* ^15^N values is greater or the same as for *δ* ^13^C values" Or "Figure 5A shows large variation in *δ* ^13^C values".

Similarly, in Figure 5B, two sequential plots of *δ* ^18^O and *δ* ^13^C values from tooth enamel can look very different depending on the scale, leading to comments such as: "Individual 1 shows larger variation in *δ* ^13^C than *δ* ^18^O values throughout the period of enamel formation".

In both cases, these comments are either inaccurate or require legitimate comparison with another sample group or *δ* value on the same scale (of the same total length in ‰ and with the same ‰ divisions). In practice, data comparisons and evaluations are always best made using statistical tests. One must also ask whether measurement uncertainty, isotopic heterogeneity of the sample matrix, and physiological effects outweigh the archaeological significance of the variation demonstrated.^64^, ^65^ This is particularly true when scales drop close to or below the measured ‰ uncertainty for *δ* ^15^N, *δ* ^13^C, and *δ* ^18^O values within a given study or laboratory (see above).

**Figure 6:**
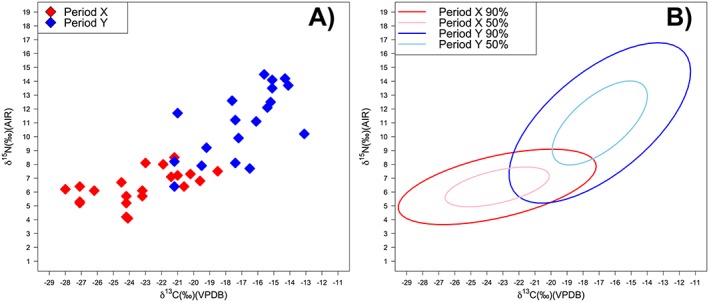
Scatterplot of *δ* ^15^N and *δ* ^13^C data for two groups overlain by Ellipses calculated at the 50% and 95% confidence intervals using the R^67^ function 'Ellipse' [Color figure can be viewed at wileyonlinelibrary.com]

It is important that all data points are displayed in graphs where possible, even if only in the supplementary information, and not just a summary representation (e.g. a boxplot or mean and standard deviation plot). This is a particularly significant problem with the growing use of ellipses to summarize archaeological *δ* parameters for a particular data group. These can be drawn using different methods and principles that should be clearly outlined. Furthermore, the confidence level of the ellipse should be stated. In single‐compound analysis of fatty acids in archaeological pottery, for example, the archaeological data are commonly plotted against 68 % confidence ellipses of modern foods corrected for the *Suess effect* (e.g. 66). As can be seen in Figure 6, a confidence ellipse of 50 % and one of 95 % can have very different relationships to the real spread of the data. This is particularly sensitive for the relatively small sample sizes that are frequent in archaeological research.

## 9 STATISTICS: THE FINAL FRONTIER

In terms of basic reporting, summary statistics such as mean or median (or both), standard deviation or interquartile range, and range should be provided where appropriate for any group of interest. Standard deviation and standard error should be used depending on the research question or point being made. Standard deviation will demonstrate how much variation there is among individual observations in a given sample, while the standard error shows how good the estimation of the mean is.^68^ As noted above, if there is concern about the homogeneity of a given sample it is also often useful to provide the *δ* values from multiple measurements on separate sample aliquots and provide the summary statistics for that comparison.

When comparing the *δ* values of different groups it should always be remembered that the phrase: "*There is a significant difference between the δ* ^*15*^ *N values of period x and period y*" is only valid if followed up by a relevant statistical test, its parameters, and full results, and would be even more appropriately phrased as: "*The null hypothesis that there are no differences in δ* ^*15*^ *N values of period x and period y can be rejected*".

An obvious difference in periods x and y might be observed from a graph of box plots or means and standard deviations where there is no overlap and can be stated as such. However, a "significant" difference is a term limited to statistical analysis and must be used accordingly.

**Figure 7:**
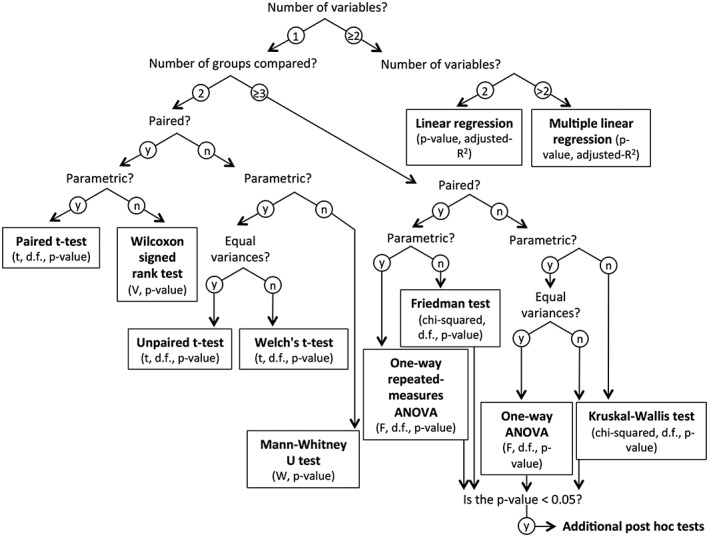
Comparison of different, commonly used statistical analyses potentially useful for evaluating *δ* value differences between groups in archaeological datasets (based on McCrum‐Gardner^70^ and Marusteri and Bacarea^71^)

It is also important that the appropriateness of the chosen statistical test is discussed.^69^ It should be clear that the data has been evaluated, either through a histogram or normality test. From this point the appropriate choice of parametric (normally distributed data) and non‐parametric test (non‐normally distributed data) should be made (see Figure 7 for a comparison of basic tests used to compare stable isotope data from groups of archaeological samples). Many archaeological scientists now utilize complex statistical models and tests. In these cases, every effort should be made to walk a non‐specialist reader (and reviewer) through the building of the model, including the reasoning as to why certain parameters have been included or not. This not only facilitates future replication but also enables more adequate evaluation of the methodology and the validity of the assumptions used.

When reporting the results of the statistical test, full information for the test chosen should be provided (Figure 7) plus the chosen significance level (typically set at or below 5 %). This then defines the boundary of a significant or non‐significant result. It is therefore not worthwhile to compare data as being more or less close to being significantly different, as that would ignore this basic statistical concept. Statistical test result tables should be fully provided where appropriate, in supplementary information if necessary. It should also be clear how the data presented was "plugged into" a given method so that the test can be replicated easily.

As discussed in section 4, a statistical method is only as good as the data that it is used to analyze. This is obvious from the development, and occasional misuse, of Bayesian mixing models that have been applied to model food source contributions to human and animal diets based on stable isotope ratios.^72^ These models quantify, as probability distributions, the contributions from food sources by considering multiple parameters (e.g. consumer and food isotopic signals, diet‐to‐consumer isotopic offsets, food nutrient concentrations, complex dietary routing mechanisms) and respective uncertainties. Here, it is essential to adequately characterize the 'true' uncertainty of 'consumer' and 'food' isotope ratios and also utilize meaningful groups or sources.

For example, a limited number of isotopic proxies, isotopic similarities among foodstuffs, and the impossibility of identifying all potential source contributions mean that identifying inputs from a particular taxon is almost impossible. Generic groupings (e.g. terrestrial plants, terrestrial animals, freshwater fish, marine fish) with associated conservative uncertainties, often around one order of magnitude larger than measurement uncertainties, are more appropriate.^73^ Diet‐to‐consumer isotopic offsets and routing mechanisms are quantified through controlled feeding experiments on humans or omnivorous mammals. The complexity of dietary routing mechanisms means that mixing models that account for separate signal contributions from multiple food nutrients towards consumer tissues are best employed in certain contexts.^74^, ^75^

Evaluation of compound‐specific data rests increasingly on classification methods and multivariate statistical approaches that are not yet widely applied in archaeology. Ecologists typically use these methods to emphasize variation and highlight strong patterns in multivariate isotopic datasets by either applying principal component analysis (PCA) or linear discriminant function analysis (LDA). While both methods are linear transformation techniques, PCA treats all data unsupervised to maximize variance among individual samples. In contrast, LDA is a supervised approach that maximizes variance among predefined groups but minimizes the variance within these groups. Hence, LDA should only be applied when additional data or observations (e.g. marine vs terrestrial) support an *a priori* classification. With PCA, one has to assess whether isotope baseline information should be factored out. In that case, center the isotope value of each compound to the mean isotope values across all compounds for each sample.

Regardless of the chosen statistical method, a central goal is to reduce the number of dimensions and identify the most informative variables. For linear transformation techniques such as PCA and LDA, we recommend reporting the significant differences between classes by employing MANOVA analyses or similar. In LDA, we suggest using leave‐one‐out cross‐validation to predict the probability of class membership of training data samples. To enhance transparency, it is good practice to report the statistical results either by describing the main outcome in the text or by including the statistical output in the supplementary information. Care should be taken in applying multivariate statistics when the number of variables considered is large relative to the number of samples. For discriminant analysis we recommend, as per Carter,^76^ that the total number of observations must be significantly greater than the number of independent variables (>5:1), and that the number of observations in the smallest group must be greater than the number of independent variables.

## 10 CONCLUSIONS

This review is not meant as a criticism directed towards any particular group of researchers, and indeed the authors have certainly been guilty of some of the above‐listed failings at some point. In the interest of best scientific practice, we have attempted to summarize the basic requirements of terminology, sampling, measurement, reporting, display, and analysis in the presentation and publication of isotope data. Much of this is dictated by IUPAC^10^ and influential members within geochemistry, mass spectrometry, and archaeological science itself, have leveled similar criticisms. However, as an increasing number of students and researchers from different academic backgrounds enter archaeological science it is beneficial to circulate these widely within an archaeology‐focused format that is as accessible as possible to this readership (including by making this article Open Access).

We have focused on bulk stable carbon and nitrogen isotope analysis of organics; bulk stable carbon and oxygen isotope analysis of carbonates; and single‐compound stable carbon and nitrogen isotope analysis on amino acids in collagen and keratin; and single‐compound stable carbon and hydrogen isotope analysis on fatty acids. However, with developments in archaeological science applications, the same principles of terminology, sampling, calibration, quality assurance and control, graphical representation, and statistical analysis will apply to the currently rarer, or more experimental, applications of bulk organic sulfur, hydrogen, and oxygen stable isotope analyses and stable hydrogen analysis of amino acids.

We request that reviewers agreeing to evaluate publications involving isotope analysis familiarize themselves with these requirements, making the appropriate critique and suggestions where necessary, so as to raise the standard of science, data production, and data availability in this ever‐expanding and advancing field.
